# A Rare Case of Guillain Barré Syndrome - Acute Facial Diplegia and Bilateral Distal Extremity Paresthesia Variant

**DOI:** 10.51894/001c.144507

**Published:** 2025-09-30

**Authors:** Yasmin Rassouli, Nahel Abugattas, Isaac Turner

**Affiliations:** 1 College of Osteopathic Medicine Michigan State University; 2 Department of Neurology Henry Ford Macomb

## 115

### BACKGROUND

Guillain-Barre Syndrome (GBS) is an acute inflammatory demyelinating polyneuropathy that can lead to rapid ascending flaccid paralysis and is often life threatening. Recognizing rare variants of GBS has become increasingly important in this setting. Prompt diagnosis and serial hemodynamic and neurologic monitoring are needed to guide treatment with either plasma exchange or intravenous immunoglobulin (IVIG).

### CASE

Here we present a rare variant of GBS causing Acute Facial Diplegia and Bilateral Distal Extremity Paresthesia in a 48-year-old female following possible viral illness three weeks prior. After extensive neurological workup which yielded positive inflammatory markers but negative imaging studies, and negative Lyme, varicella zoster, and Sjogren’s serologies as well as negative antinuclear antibodies and oligoclonal bands on cerebrospinal fluid analysis, we were left with a diagnosis of GBS. Treatment with IVIG (10% IV solution 45g every other day for 3 days) was initiated.

### DISCUSSION

GBS is a common cause of facial diplegia. Facial diplegia with paresthesias is a rare localized variant of GBS, characterized by simultaneous facial diplegia, distal paresthesias, and minimal or no motor weakness. Currently, most studies mention only a handful of cases of the Acute Facial Diplegia and Bilateral Distal Extremity Paresthesia variant. Despite the incidence of this GBS variant being less than 1%, strong clinical examination along with comprehensive neurological workup aided in reaching this final diagnosis. The patient was started on IVIG therapy and slowly began showing improvements by the third day of treatment.

### CONCLUSION

Diagnosing rare variants of GBS has proven difficult. This case highlights the complexity of ruling out other etiologies that could lead to these neurologic sequelae seen in the Acute Facial Diplegia and Bilateral Distal Extremity Paresthesia variant of GBS and the importance of comprehensive patient care and symptom management.

